# Dynamic Gene Expression in the Human Cerebral Cortex Distinguishes Children from Adults

**DOI:** 10.1371/journal.pone.0037714

**Published:** 2012-05-30

**Authors:** Kirstin N. Sterner, Amy Weckle, Harry T. Chugani, Adi L. Tarca, Chet C. Sherwood, Patrick R. Hof, Christopher W. Kuzawa, Amy M. Boddy, Asad Abbas, Ryan L. Raaum, Lucie Grégoire, Leonard Lipovich, Lawrence I. Grossman, Monica Uddin, Morris Goodman, Derek E. Wildman

**Affiliations:** 1 Center for Molecular Medicine & Genetics, Wayne State University School of Medicine, Detroit, Michigan, United States of America; 2 Departments of Pediatrics and Neurology, Wayne State University School of Medicine, Detroit, Michigan, United States of America; 3 Department of Computer Science, Wayne State University, Detroit, Michigan, United States of America; 4 Department of Anthropology, The George Washington University, Washington DC, United States of America; 5 Fishberg Department of Neuroscience and Friedman Brian Institute, Mount Sinai School of Medicine, New York, New York, United States of America; 6 Department of Anthropology, Northwestern University, Evanston, Illinois, United States of America; 7 Department of Anthropology, Lehman College & The Graduate Center, City University of New York, Bronx, New York, United States of America; 8 Department of Anatomy & Cell Biology, Wayne State University School of Medicine, Detroit, Michigan, United States of America; 9 Center for Molecular Medicine & Genetics and the Department of Obstetrics & Gynecology, Wayne State University School of Medicine, Detroit, Michigan, United States of America; Nathan Kline Institute and New York University School of Medicine, United States of America

## Abstract

In comparison with other primate species, humans have an extended juvenile period during which the brain is more plastic. In the current study we sought to examine gene expression in the cerebral cortex during development in the context of this adaptive plasticity. We introduce an approach designed to discriminate genes with variable as opposed to uniform patterns of gene expression and found that greater inter-individual variance is observed among children than among adults. For the 337 transcripts that show this pattern, we found a significant overrepresentation of genes annotated to the immune system process (pFDR≅0). Moreover, genes known to be important in neuronal function, such as brain-derived neurotrophic factor (*BDNF*), are included among the genes more variably expressed in childhood. We propose that the developmental period of heightened childhood neuronal plasticity is characterized by more dynamic patterns of gene expression in the cerebral cortex compared to adulthood when the brain is less plastic. That an overabundance of these genes are annotated to the immune system suggests that the functions of these genes can be thought of not only in the context of antigen processing and presentation, but also in the context of nervous system development.

## Introduction

Many of the behavioral traits that distinguish humans from other species are molded throughout development by an exceptional capacity to incorporate experience and learning into the production of culture [1]. This ability to learn results from changes that occur in the organization of the brain as the result of experiences. The cerebral cortex is continually remodeled in response to various molecular signals, as well as environmental stimuli [2]. This phenotypic plasticity in the brain can occur at multiple levels, ranging from large-scale remapping of cortical areas to more subtle strengthening or weakening of synaptic connections [3]. Indeed, an enhanced capacity for such plasticity may be important in the development of evolutionarily distinct aspects of human cognition [4]–[6]. While a certain degree of plasticity is exhibited throughout one's lifetime, the juvenile brain undergoes a greater degree of cortical remodeling than does the adult brain [7], [8]. Determining the molecular underpinnings that enable this heightened cortical plasticity is fundamental for uncovering the mechanisms subserving the development of human brain function. A particularly promising avenue of research in this area focuses on identifying how the expression of genes changes throughout development [9]–[11]. Such changes in the regulation of gene expression may have significantly influenced human brain evolution [12].

Regulation of gene expression in the brain may be one way the developing nervous system is able to exhibit plasticity in response to changes in the environment. This phenotypic plasticity likely results from modifications to neuron morphology and synaptic connections as well as to the surrounding cells (e.g., oligodendrocytes, microglia, astrocytes) that participate in neuron function. As a result, a reflection of phenotypic plasticity in the brain may be observed by measuring variability in gene expression in samples of cerebral cortex tissue taken from individuals of different ages. In the current genome-wide study we sought to clarify whether mRNA expression in children is more dynamic and variable than gene expression in adult brains. We reasoned that those genes for which cortical expression is more variable across individuals during childhood and early adolescence, compared to adults, could be considered candidates for involvement in neuronal and synaptic plasticity. To explore these questions, we used microarray techniques to examine gene expression in surgically-resected human cerebral cortex tissue in individuals ranging in age from less than one year to 53 years of age. Using these data, we develop an approach designed to discriminate the degree to which gene expression is variable across individuals of the same age class, and then test for age related differences in the degree of this variability.

## Results

### Genes with Greater Variance in Expression During Childhood

**Figure 1:**
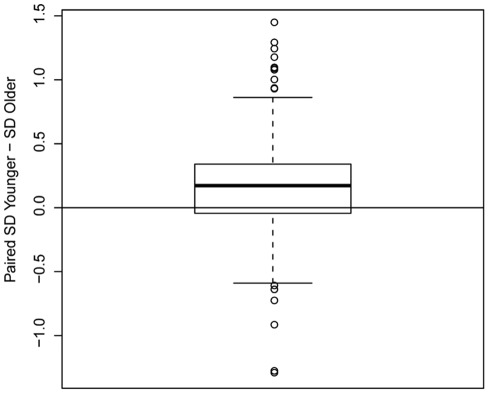
Paired differences between the standard deviation of genes in children vs. adults. The boxplot is made from for all 1095 probes with highest variance across all samples. Positive values indicate larger standard deviation (SD) in the younger group (children<15 years), whereas negative values indicate larger standard deviation in the older group (adults≥15 years). The median (heavy black line) represents the point at which 50% of the data are greater than (above the line) or less than (below the line) this value. The upper quartile (open box above the median) represents the 25% of the data greater than the median. The lower quartile (open box below the median) represents the 25% of the data less than the median. Note that 71% of the probes have greater standard deviation in the younger group. The maximum (above the upper quartile) and minimum (below the lower quartile) values excluding outliers are also shown. Outliers are drawn as open circles.

We sought to identify which genes showed expression patterns in the cerebral cortex that are variable across individuals and to then evaluate whether there were age differences in the degree of this variability. To test this, 20,678 well-curated microarray probes were sorted by variance in expression level across all samples, regardless of age, and the probes with greatest variance (5%, n = 1095) were retained for further analyses. We then divided the 37 samples into two age groups [younger (<15 years old) and older (≥15 years old)] and compared the variance between groups for each gene. A cutoff of 15 years was used to distinguish older individuals with adult levels of cortical glucose uptake [6] and synapse organization and density [13], [14] from younger individuals. We then calculated the expression variance in each group (younger and older) and tested whether the ratio of the variance of the younger group to the older group was greater than 1 for each probe using an F-test. Of the 1095 most-variable probes, 71% showed significantly greater variance during childhood than during adulthood [p<1×10^−16^ (Wilcoxon signed-rank test); Figure 1]. Of these, a total of 337 probes (Dataset S1) corresponding to 302 annotated genes showed significantly greater expression variance during childhood (pFDR<0.25; 93 probes with FDR<0.1).

**Figure 2:**
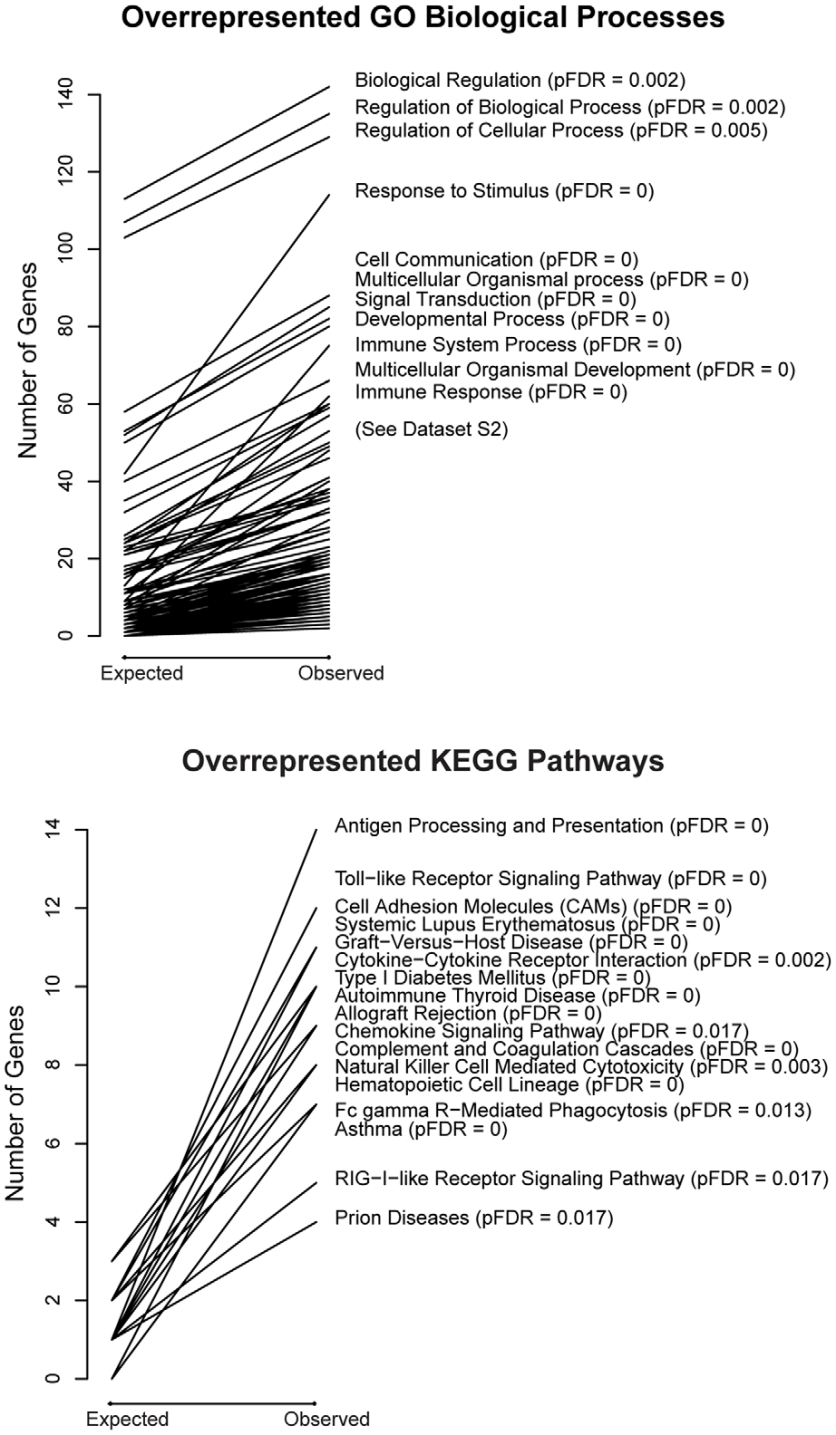
Gene Ontology and KEGG pathway analyses (**reference  =  all genes**)**.** Gene Ontology Biological Process (GO_BP) and KEGG pathway analyses for probes with greater variance in childhood than in adulthood using as reference all genes called present on the array. The *expected* number of genes is the number of genes predicted for this term by random chance. The *observed* number of genes is the number of genes actually present in our dataset for this term. For example, in this context we would expect by random chance to see 13 genes annotated to the GO_BP term ‘immune system process’ (GO:0002376). Instead, we observed 75 genes annotated to this term (pFDR  = 0). The steepness of the slope of each line reflects statistical significance with steeper lines having smaller pFDR values. Those categories with the greatest slope (pFDR  = ≤0.02) are labeled in this figure. All 339 GO_BP terms and 19 KEGG pathways that met our enrichment criterion of pFDR ≤0.1 can be found in Dataset S2. Additional GO (Molecular Process and Cellular Component) data and plots can be found in Dataset S2 and Figure S1 respectively.

**Figure 3:**
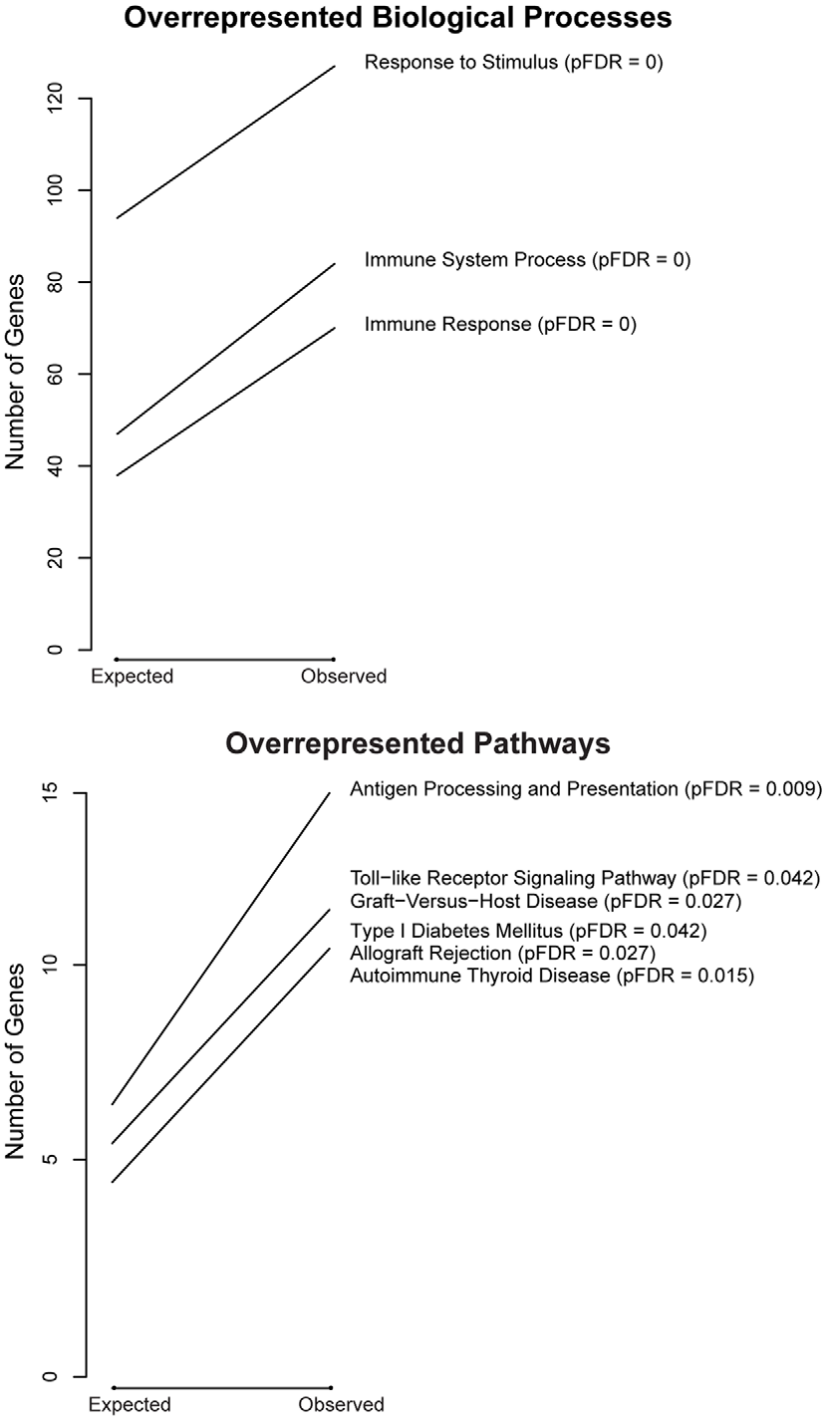
Gene Ontology and KEGG pathway analyses (**reference  = 1095 probes with greatest variance**)**.** GO and KEGG pathway analyses for probes with greater variance in childhood than in adulthood using as reference the 1095 (∼5%) genes with highest variance across all samples. The *expected* number of genes is the number of genes predicted for this term by random chance. The *observed* number of genes is the number of genes actually present in our dataset for this term. For example, in this context we would expect by random chance to see 47 genes annotated to the GO_BP term ‘immune system process’ (GO:0002376). Instead, we observed 84 genes annotated to this term (pFDR  = 0). The steepness of the slope of each line reflects statistical significance with steeper lines having smaller pFDR values. Those categories with the greatest slope (pFDR  = ≤0.02) are labeled in this figure. All 10 GO_BP terms and 6 KEGG pathways that met our enrichment criterion of pFDR ≤0.1 can be found in Dataset S3. Molecular Function (MF) analyses did not meet enrichment criteria. Cellular Compartment (CC) has one term that met our criterion (MHC protein complex; pFDR  = 0.09; see Dataset S3).

Gene ontology analyses were used to help identify overrepresented gene annotation terms in this list of 337 probes. These analyses revealed that the most overrepresented biological processes, when compared to all genes present on the array, were immune system process (BP_GO:0002376) and immune response (BP_GO:0006955) (Figures 2 and S1 and Dataset S2). We also conducted gene ontology and pathway analyses using as the reference list the subset of 1095 probes with greatest variance regardless of age. With this analysis we found further enrichment for particular ‘immunity-related’ terms and pathways (Figure 3 and Dataset S3).

To examine the effect of having multiple brain regions included in the childhood dataset, these analyses were repeated using only samples collected from the temporal lobe. The resulting list of significant probes and the original list of 337 probes have 86% overlap (Dataset S1). As a result, similar gene ontology terms and pathways were enriched whether all samples or only the temporal cortex samples were used.

**Figure 4:**
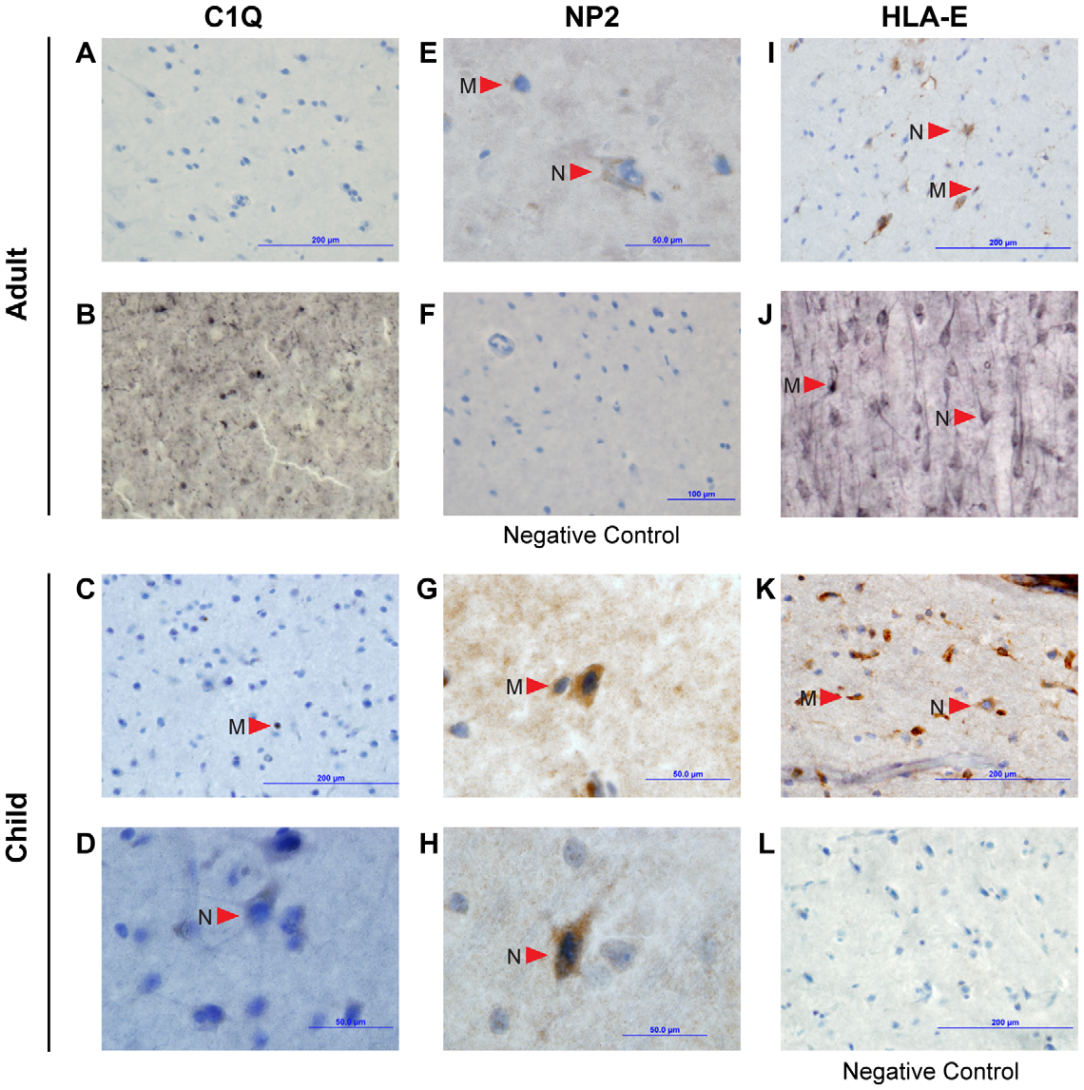
Immunohistochemistry evidence for expression of ‘immunity-related’ genes in neurons and glial cells. Immunohistochemistry showing the protein expression of C1Q, NP2 and HLA-E in nondiseased human glial cells and neurons. Arrows denote the location of microglia (M) and neurons (N). Images A–B show C1Q staining in frozen temporal lobe sections of two adults. Images C–D show C1Q staining of microglia and neurons, respectively, in frozen temporal lobe sections of a child. Image E shows NP2 staining of microglia and neurons in frozen temporal lobe sections of an adult. Image F is a negative control of the adult frozen temporal lobe tissue. Images G–H show NP2 staining of microglia and neurons, respectively, in paraffin embedded temporal lobe section of a child. Images I–J show HLA-E staining of both microglia and neurons in frozen temporal lobe sections of two adults. Image K shows HLA-E staining of microglia and neurons in a frozen temporal lobe section of a child. Image L is a negative control of the frozen temporal lobe section of the child. We found evidence for the expression of all three proteins in both cell types of the child. However, in the adult, NP2 and HLA-E were present in both cell types but there was no evidence of C1Q expression.

Our gene expression analyses were based on tissue homogenates, and thus we sought to determine the cellular localization pattern of proteins that showed a more variable mRNA expression pattern in children. Specifically, we used immunohistochemistry to determine the cellular localization for three proteins encoded by genes that were found to be more variable in their expression in childhood (HLA class I histocompatibility antigen, alpha chain, HLA-E; a subcomponent of the classical pathway of complement activation, C1q; and Neuronal pentraxin-2, NP2). We found that all three proteins are expressed in microglia and/or neuronal cells in human temporal tissue (Figure 4). In addition, we found evidence for the expression of all three proteins in both cell types in a temporal cortex sample from a child. HLA-E expression was detected in microglia and neurons of both adults examined. Expression of NP2 was found in both microglia and neurons of one adult examined. There was no evidence; however, of C1q expression in either adult (Figure 4). The absence of C1q immunoreactivity in cells of the cerebral cortex of adult humans was further confirmed in an additional four individuals (data not shown).

Recent studies have identified a large number of developmentally regulated genes [9], [11]. One possible explanation for the greater variance we observed among children is that expression of these genes is developmentally regulated. To test for this we used quadratic regression to examine the expression levels of the 302 genes that have evidence for a wider expression variance in childhood as a function of age (Dataset S1). We found that only 1 of the 302 genes is differentially expressed as a function of age (*NQO1*; pFDR = 0.18), the remaining 301 are not (pFDR ≥0.25). Thus, we are confident that childhood inter-individual variation rather than developmental trajectories explains the observed results.

To evaluate whether the greater gene expression variance among children and overrepresentation of genes annotated to the immune response is a result of using RNA derived from control (normal) tissue harvested from surgically resected samples, these analyses were repeated using expression data derived from postmortem tissues taken from individuals having no history of psychiatric or neurological complaints [GEO accession: GSE13564 [9]]. Of the 1020 genes representing the 5% of genes with greatest expression variance across all samples, 78% showed higher variance in the younger group (<15 years) compared to the older group (≥15 years) (p<2.2×10^−16^; Figure S2). Of these 1020 genes 529 were significant (pFDR<0.25) in this analysis. The list obtained by intersecting the significant genes in the variance analysis based on the Harris et al. dataset included 34 genes (Dataset S4); by random chance we would have expected to find approximately 55 overlapping genes between the two studies. Thus, specific genes involved in plasticity show variation between the two studies. Biological processes related to immunity (e.g. BP_GO:0006955, GO:0002252, GO:0050776) and nervous system development (BP_GO:0007399) were enriched (p<0.01; Dataset S5) in the list of 529 significant genes, which was similar to what was observed based on current study data.

### Validation of Microarray Data Using qPCR

**Figure 5:**
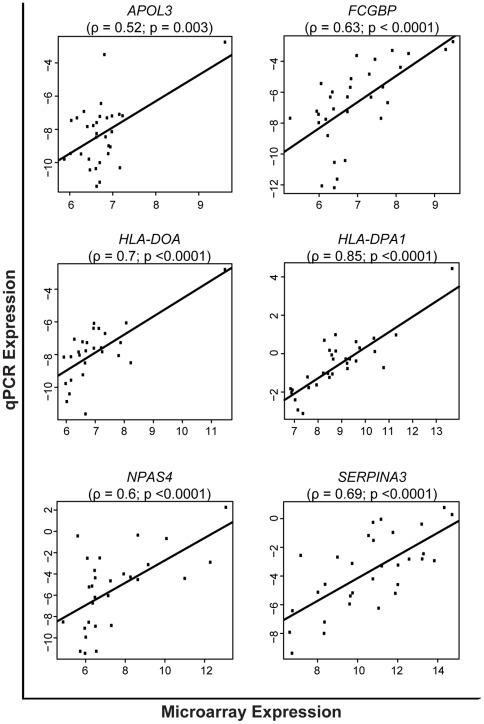
Test of the correlation between microarray and qPCR data. Y-axis shows mRNA expression levels [–DCt values (Ct reference–Ct target)] derived from qPCR experiments whereas the X-axis shows the log2 normalized microarray expression signal intensities. Correlations were considered significant when p<0.05. ρ = correlation coefficient.

We chose for validation fourteen probes annotated to genes with greater variance in expression during childhood (*HLA-DOA*, *APOL3*, *LEP, C1QC, CHURC1, HLA-DOA, HLA-E, NPTX2, PCDH17, HLA-DPA1, SERPINA3, NPAS4, IL8* and *FCGBP*). The qPCR data of six of these probes *(APOL3*, *HLA-DOA, HLA-DPA1, SERPINA3, NPAS4,* and *FCGBP)* were in agreement with microarray findings (Figure 5). The genes that did not validate, failed because of 1) poor Illumina probe annotation; and 2) the microarray results may be more sensitive in detecting variance than is qPCR.

## Discussion

Among transcripts showing the greatest variance in expression levels, we found a significantly higher number of genes (n = 302) in which expression variability among individuals was greater in samples collected in children and young adolescence than those collected in adults. These data provide compelling evidence for greater variance in the expression of genes in the cerebral cortex earlier in development. We note that this variance is associated with the general greater overall plasticity during childhood [7], [8]. Determining how and if such inter-individual variance in the transcriptome relates to plasticity among neurons and at the synapse and how it manifests as phenotypic or behavioral variability is of fundamental importance for uncovering the biological mechanisms underlying human brain development. These genes and their related pathways make compelling candidates for further study of nervous system development and cognition, and they also reinforce a role for system-wide plasticity and adaptation in the nervous system. Strikingly, many of these genes are known to function in the immune system. These data suggest many genes traditionally considered to be ‘immunity-related’ are not only expressed in the brain but also show greater variance in their expression levels at a developmental stage characterized by heightened neuronal and synaptic plasticity.

### Convergence of Neurologically Expressed Responses in Adulthood

Many of the 302 genes (e.g., brain-derived neurotrophic factor) with more dynamic patterns of gene expression during childhood already have documented roles in the nervous system. When we intersect our list of 302 genes with phenotype data (largely derived from knock out experiments in mice [15]) compiled by Mammalian Genome Informatics [16], we find 238 of these genes have orthologous mouse genes represented in the phenotype database. Half of all protein-coding genes with a mammalian phenotype (MP:0000001) show some sort of nervous system phenotype (3,011 of 6,406). Only 66 of the 238 variable orthologous human genes have a noted neuronal phenotype (Mammalian Phenotype IDs: MP:0003631 and MP:0005386; Dataset S6), in mice. This is a significant underrepresentation (2 tailed p≤0.0001, Fisher's Exact Test). Future work will be necessary to understand whether the neuronal function of these human genes represents an evolutionary divergence from mouse genes.

That we detect a high degree of inter-individual variability in the regulation of these transcripts during childhood may reflect the fact that this is a developmental stage characterized by increased neuronal plasticity. From both an immunological [17] and neurological [7] perspective, children have a more naïve and less established response to novel environments when compared to adults. Both the child's nervous and immune systems develop in response to stimuli [18], and because children are initially exposed to different stimuli at different times, we might speculate that what happens to forge a connection between a stimulus and a response in one child may be different than what happens to forge that same connection in another. However, as children age, the efficiency and speed of the response (both neurological and immunological) to stimuli grows, and during adulthood these responses may converge regardless of the nature of their initial exposures [19], [20]. This could result in more targeted, yet less plastic, responses to environmental stimuli during adulthood. We suggest that greater variability in the expression of these ‘response genes’ in the brain during childhood could reflect a developmental period during which neurological responses are being established and the brain is more plastic [7]. We might speculate on the mechanistic level that during childhood the brain is responding to stimuli with more *ad hoc*, less fixed signaling and biochemical pathways. Although this may be necessary during childhood, such variability is energetically costly to maintain [6], [21] and should be selected against when an effective response is already present. Therefore, just as exposure converges between individuals during adulthood, we observe similar convergence at the transcript level of these genes between adults. Importantly, we found a very similar pattern in two independent and distinct datasets.

### Immune System Genes in the Normal, Healthy Brain

Of the 302 genes identified as having greater variance among children, 84 were annotated to the immune system. The central nervous system (CNS) has traditionally been considered immune privileged; a system in which white cells and plasma proteins tolerate the introduction of alloantigens rather than initiating an immune response. However, similarities between the immune and central nervous systems have previously been suggested [22], [23] and recent research shows increased evidence of cross-talk between the two systems [2], [24]–[26] as well as shared mechanisms underlying similar structures and functions [18]. Although ‘immunity-related’ proteins such as IL-1, IL-6 and TNF [27]–[29], CXCL12/CXCR4 [30]–[32], C1q and C3 [33], [34], and the major histocompatibility complex class I (MHCI) family [35]–[37] have been shown to function in the healthy CNS, the current research suggests that this is only a subset of ‘immunity-related’ genes expressed in healthy brain tissue. Our dataset of 84 genes annotated to ‘immune system process’ (BP_GO:0002376; Dataset S3) includes genes implicated in the classical complement cascade (e.g., *C1QB, C1QC, C2, C5AR1*), chemokine signaling and cytokine-cytokine interactions (e.g., *CCL26, CCL5, CCR1, CXCL10, IL6, IL8, LEP, OSM, TNFSF10, TNFSF13B, WAS*, and *DOCK2*), MHC class II receptor activity (e.g., *HLA-DMA, HLA-DOA, HLA-DPA1, HLA-DRA, HLA-DRB3*, and *HLA-DRB4*), MHC class I receptor activity (e.g., *HLA-A, HLA-B, HLA-E,* and *HLA-H*), cell adhesion (e.g., *CD86, ITGAL, ITGB2, SPN,* and HLA molecules), and Toll-like receptor signaling (e.g., *CD14, CD86, CCL5, CXCL10, IL6, IL8, LY96, TLR7*). Although many of these genes have traditionally been considered ‘immunity-related,’ it is important to note that, unlike their roles in the immune system, the processes discussed here (e.g., MHCI receptor activity, cell adhesion) appear to function in the brain free from injury, disease and exposure to pathogens [38]. Both glial cells (e.g., microglia) and neurons appear capable of expressing these genes in the central nervous system under normal conditions [35], and additional work is needed to decipher whether glial or neuronal functions contribute to the pattern of gene expression described here.

Blood contamination in cortical tissue could confound our gene expression result, especially in the case of immune related genes. Thus, we provide evidence, using immunohistochemistry, that HLA-E is present in both glial cells and neurons of a child's brain as well as two adult brains (Figure 4). To our knowledge, the expression of this protein has previously only been characterized in tumor cells [39] and in trophoblast cells [40]. We also found evidence for protein expression of C1q and NP2 in both cells types of the child brain, NP2 expression in both cell types of the adult brain but no evidence of expression of C1q in two adult brains. This finding is consistent with studies suggesting an interaction between NP2 and C1q in mice [34]. Our findings suggest the potential exists for a similar interaction in the human brain, but this interaction may be limited to the younger, more plastic cortex. That we did not detect expression of C1q in the adult brain provides evidence that this protein plays a more prominent role in the developing rather than the adult brain. Further work is necessary to determine the relationship between mRNA and protein expression of C1q.

Previous studies have demonstrated differential expression of MHCI gene and protein expression in regions of the central nervous system undergoing activity-dependent plasticity [36], [37], [41]–[45]. Whereas the exact mechanisms underlying MHCI's role in plasticity are not yet known, a growing body of evidence points to MHCI protein involvement in selective maintenance or elimination of synapses throughout normal brain development and cell-cell communication [25], [36], [37] as well as a role in neurotransmission at the synapse [44]. In addition, it was recently shown that neuronal MHCI has the ability to modulate NMDA receptor function and NMDA-induced AMPA receptor trafficking, which suggests a role for MHCI in NMDAR-dependent synaptic plasticity [35]. The authors note that because MHCI levels vary during development and are activity dependent, changes in MHCI levels may provide a mechanism for developmental changes in plasticity and synaptic activity in the brain [35]. In children in the present study, MHCI genes like *HLA-A, HLA-B*, and *HLA-E* are expressed at different levels in different individuals. It is tempting to speculate that such ‘immunity-related’ genes expressed by neurons may encode proteins that can initiate processes important for nervous system development.

### Is the dynamic pattern of gene expression among children uniquely human?

When considering the evolution of the human brain in particular we propose humans may exhibit a more prolonged period of adaptive mRNA expression variability during childhood. Humans have a longer period of juvenile development in comparison to other primates [46], [47]. During this time the cerebral cortex consumes nearly twice the amount of glucose as observed during adulthood [21] and there are extensive changes to synaptic organization and density [13], [14]. Our data suggest that another characteristic of the human cerebral cortex is greater inter-individual variance in gene expression earlier in development. Because we observe this pattern in tissue samples that are heterogeneous in cellular composition, we suggest this variance relates to overall phenotypic plasticity in the brain but further work is needed to distinguish the relative contribution of each cell type to this transcriptional variability in childhood. Genes that show this pattern may contribute to phenotypic plasticity in the brain and serve as mediators between the environment and genome.

Testing if this prolonged period of adaptive gene expression is unique to humans will require examining gene expression throughout development in nonhuman primates. One promising potential avenue for pursuing this aim is to examine regulatory elements of the 302 genes identified in this study across nonhuman primates. Those elements fixed in modern human populations but derived from the ancestral condition may point to human specific features. Additionally, examining variance in gene expression in nonhuman primate cortical tissue would provide a more comparative context and allow us to infer if the pattern that we observe in these human samples is evolutionarily derived. Good candidate species for this type of study include chimpanzees and rhesus macaques. Unlike humans and chimpanzees, macaques have a much shorter period of juvenile development [46], [47] and when compared to humans, and glucose consumption levels also peak earlier in development than in humans [48]. Although many of these same genes may be expressed in nonhuman primate cortical tissue and would show similar degrees of expression variance in younger vs. older individuals, we would suggest that the period of greater expression variance would be greatly reduced in species with shorter periods of brain development. Understanding these processes from an evolutionary perspective may provide clues to the origin and refinement of human cognitive features.

## Materials and Methods

### Samples

Samples (Table S1) were derived from surgically resected control tissues (e.g., electrophysiologically inactive) that were removed from patients during surgery for brain disorders (e.g., intractable seizures). Written informed consent was obtained from all patients involved in the study; in the case of children, consent was obtained from authorized legal representatives (e.g., parents/legal guardians). Procedures for obtaining consent were approved by the human investigation committee. Race and/or ethnicity were self-identified, and patients were then categorized according to National Science Foundation established criteria. The research described in this publication was reviewed and approved by the Human Investigation Committee (HIC) at Wayne State University (HIC# 071608MP4X) and was found to present no greater than minimal risk to human subjects.

Cerebral cortex tissues taken primarily from the temporal lobe (Table S1) were flash frozen in dry ice and stored at −70°C. Tissue samples were homogenized in TRI Reagent (Applied Biosytems/Ambion, Austin, TX). RNA extraction was completed using the TRIzol protocol (Invitrogen, Carlsbad, CA) or the MagMax-96 for Microarray kit (Applied Biosystems/Ambion) following the manufacturers' instructions. The TURBO DNase treatment (Applied Biosytems/Ambion) or RNeasy kit in conjunction with the RNase-Free DNase Set (Qiagen, Valencia, CA) was used to further purify the initial RNA isolation according to the manufacturer's recommendations. The DNA-free RNA isolations with ABS 260/280 ratios above 1.7 (Nanodrop 1000; Thermo Scientific, Wilmington, DE) and two distinct peaks representing the 18S and 28S ribosomal RNA and minimal background banding (Agilent Bioanalyzer 2100; Santa Clara, CA), were selected for analysis. Gene expression data were collected using a genome-wide microarray [Illumina Human HT-12v3 chips using the Illumina BeadChip platform (Illumina, San Diego, CA)] by the Applied Genomics Techology Center (AGTC; Wayne State University, Detroit, MI) following Illumina's protocol in the TotalPrep-96 RNA Amplification Kit (Applied Biosytems/Ambion, Austin, TX) for labeling and the Whole-Genome Gene Expression with IntelliHyb Seal for the hybridization, wash, and stain (Illumina, San Diego, CA).

### Data Preprocessing

Illumina BeadStudio (V.3) was used to process the arrays and obtain background corrected intensity values for all 48,803 probes in 40 arrays (corresponding to 37 unique brain samples) as well as the probe detection p-values. Only probes with a detection p-value ≤0.1 in at least half (16) of the total number of unique individuals sampled were retained for future analyses. This filtering step resulted in a total of 20,678 probes for our variance analyses. All values in the background corrected expression matrix were offset by adding a constant so that the smallest intensity value equaled 1.0 in order to allow further log transformation of the data. Expression values were then log_2_ -transformed and quantile-normalized [49]. A final preprocessing step averaged over the expression values for each of the 3 samples run in duplicate.

### Variance in Expression Level by Age Group

The log_2_ gene expression data for each gene was first adjusted for the SEX variable by subtracting from all male sample expression levels the male average expression level and from all female sample expression levels the female average expression level. The variance of each probe was determined from these SEX adjusted data and then probes were sorted from largest to smallest variance and the top ∼5% (1095 probes) were retained for further examination. We chose to pre-filter the variant transcripts to be sure we were capturing those transcripts with the greatest overall variance among individuals. This pre-filtering step allowed us to filter out thousands of transcripts with low to nonsignificant levels of inter-individual variance and to determine, with greater statistical power, if the most variant transcripts exhibit 1) greater variance in kids, 2) greater variance in adults, or 3) if the most variant transcripts were equally variant across all individuals.

Samples were divided into two age groups: children or younger individuals (<15 years old; 29 individuals) and adults or older individuals (≥15 years old; 8 individuals). A cutoff of 15 years was chosen because cortical glucose uptake has reached adult levels [6] and changes to synapse organization and density are approaching adult levels at this age [13], [14]. Expression variance was computed in each group and we tested whether the ratio of the variance of younger individuals to older individuals was greater than 1 for each probe with an F-test. The nominal p-value derived from the F statistic accounts for the different number of degrees of freedom in the estimation of the variance of each group. A Wilcoxon signed-rank test was used to determine whether the top 5% of probes with the largest variance had greater variance in younger individuals. Gene ontology and pathway analyses were conducted for the probes (Dataset S1) with significantly greater expression variance during childhood using as reference list both a) the list of all probes called present and b) the list of 5% most varying probes.

In order to test that the variance analysis was not simply identifying genes that are differentially expressed as a function of childhood age only (i.e. developmentally regulated changes in gene expression), we used a quadratic regression approach to examine the relationships between gene expression levels and age. We employed a linear model that assumes a quadratic relationship between expression levels (log-transformed) and age while adjusting for sex and potential for developmental delay. An F-test was used to calculate a p-value for each probe representing the probability that the expression level is not significant for age. These p-values were corrected for multiple hypothesis testing and nominal as well as pFDR values for all 337 probes identified as having greater expression variance among children are reported in Dataset S1.

In order to examine the effect of having tissue derived from surgical patients, these analyses were repeated using a previously published dataset [9] with expression data derived from postmortem tissue (GEO accession: GSE13564). All 42 individuals from this dataset with age information were used in this analysis. The raw data was preprocessed using the RMA algorithm [50] implemented in the *affy* package [51] of Bioconductor, which included background correction, quantile normalization and summarization of probe intensities into one value per probeset and sample. Probesets not detected present (or marginally present) in at least half of the samples were discarded from further analyses. The present and marginal present calls were obtained using the *affy* package. In addition, probesets that could not be mapped to a valid Entrez ID were also discarded from further analyses. The difference in gene variance between age groups (<15 and ≥15 years) were performed as described above except that the sex adjustment step was skipped since there was no gender data available for this dataset.

### Immunohistochemistry

Human, nondiseased, surgically resected, temporal cortex tissues were obtained from 2 male subjects, one representing the younger group (<15 years) and one representing the older group ≥15 years). The resected temporal tissue was fixed in 4% freshly depolymerized paraformaldehyde in 0.1 M phosphate buffer saline pH 7.4 (PBS) at 4°C for 4 days. The tissue was rinsed in PBS for 30 minutes, 3 times on a rocking device and then divided into two halves and subsequently immersed in a 15% sucrose solution in PBS and placed at 4°C overnight. The samples then were placed in 30% sucrose in PBS for several days at 4°C until the tissue sank in the solution. Cryoblocks were made using Optimum Cutting Temperature compound (OCT) and stored at −80°C. With the aid of a cryomicrotome, 6-µm thick frontal sections were cut from one half of each cryoblock at −20°C to −26°C, mounted on charged glass slides and stored at −80°C for future use.

Immunohistochemistry was performed using mouse monoclonal antibodies against HLA-E (Abcam, Cambridge, MA Clone MEM-E/02, IgG1, dilution 1∶20), goat polyclonal antibody against a peptide mapping near the N-terminus of NP2 of human origin (genetic locus *NPTX2*, Santa Cruz Biotechnology, Santa Cruz, CA, dilution 1∶25) and rat monoclonal antibody C1q (7H8, Abcam, Cambridge, MA, 1∶10 dilution).

All samples were air dried and rehydrated. Immunohistochemical tissue labeling was performed using the Discovery Immunohistochemistry Auto-System (Ventana). Cell conditioning, pretreatment and blocking of peroxidases were performed according to standard protocols.

An additional, nondiseased, postmortem obtained temporal cortex sample was also stained following similar methods to those above. Prior to immunostaining, sections were rinsed thoroughly in PBS and pretreated for antigen retrieval by incubation in 10 mM sodium citrate buffer (pH 3.5) at 37°C in an oven for 30 minutes. Sections were then rinsed and immersed in a solution of 0.75% hydrogen peroxide in 75% methanol to eliminate endogenous peroxidase activity. After rinsing again, sections were incubated in the primary antibody, HLA-E (same as above, but 1∶100 dilution) or rabbit polyclonal C1QC antibody (Abcam, Cambridge, MA, 1∶100 dilution) diluted in PBS with 2% normal horse serum and 0.1% Triton X-100 detergent for approximately 24 hours on a rotator at 4°C. After rinsing in PBS, sections were incubated in biotinylated anti-rabbit IgG (1∶200 dilution, BA-2000, Vector Laboratories, Burlingame, CA) and processed with the avidin-biotin-peroxidase method using a Vectastain Elite ABC kit (pk-6100, Vector Laboratories). Sections were rinsed again in PBS, followed by a rinse in sodium acetate buffer. Immunoreactivity was revealed using 3,3′-diaminobenzidine and nickel enhancement.

### qPCR Validation

Validation of mRNA expression patterns was performed by qPCR on three genes of interest (*HLA-DOA, APOL3,* and *LEP*) using the Taqman gene expression assays available from Applied Biosystems (ABI). In order to account for the slight bias of younger individuals in the microarray analysis, 10 samples from individuals ≥15 years old and 1 sample from <15 years old were added to the 36 (1 sample lacked sufficient RNA for validation) for validation by qPCR. A total of 800 ng of RNA from each sample were synthesized into complementary DNA (cDNA) using Superscript III First-Strand Synthesis system (Invitrogen). qPCR was performed using the Stratagene (Santa Clara, CA) MX3000P real-time machine and ABI's Taqman 2X Universal PCR master mix (2X), No AmpErase UNG. The reference gene human *RPLPO*, a standard endogenous control from ABI's Taqman gene expression assays, was multiplexed with the target gene for every cDNA template as a quality control measure. All samples were run in triplicate. We also examined additional genes of interest *C1QC, CHURC1, HLA-DOA, HLA-E, NPTX2, PCDH17, HLA-DPA1, SERPINA3, NPAS4, IL8, FCGBP*, respectively, using the conditions above except the assays were run on an Applied Biosystems 7500 Fast Real-Time PCR System. Average Ct value was used for each sample. The –DCt values (Ct target – Ct reference), which are surrogates for log (base 2) gene expression were analyzed as described above.

### Probe Annotation and Gene Ontology Mapping

When an Illumina probe lacked gene annotation information we mapped the probe sequence to the human genome (Feb. 2009 GRCh37.hg19) using the UCSC Genome Bioinformatics Site Blat tool and chose the best sequence match. For all ontology analyses GO mappings were based on data provided by Gene Ontology (ftp://ftp.geneontology.org/pub/go/godatabase/archive/latest-lite/) on 2009-Aug 30 and Entrez Gene (ftp://ftp.ncbi.nlm.nih.gov/gene/DATA) on 2009-Mar 11. Onto-Express [52] was used to examine relationships between GO terms.

### Software Tools Used in Analyses

The R statistical language and environment [53] using specialized bioinformatics packages available via the Bioconductor project (www.bioconductor.org) was used for all data analyses. Bioinformatics packages used included limma, preprocessCore, marray, GOstats, KEGG.db, GO.db.

### Data Deposition

Microarray image files collected for this project are MIAME (Minimum Information about a Microarray Experiment [54]) compliant and have been deposited in the National Center for Biotechnology Information's Gene Expression Omnibus (GEO) data repository under the series accession number GSE37721.

## Supporting Information

Figure S1 **Gene Ontology analyses.** GO term (Molecular Function and Cellular Component) analyses for probes with greater variance in childhood than in adulthood using as reference all genes called present on the array. The *expected* number of genes is the number of genes predicted for this term by random chance. The *observed* number of genes is the number of genes actually present in our dataset for this term. For example, in this context we would expect by random chance to see >1 gene annotated to the GO_MP term ‘MHC class II receptor activity’ (GO:0032395). Instead, we observed 5 genes annotated to this term (pFDR = 0). The steepness of the slope of each line reflects statistical significance with steeper lines having smaller pFDR values. Those categories with the greatest slope (pFDR  = ≤0.05) are labeled in this figure. All 26 GO_MP terms and 30 CO_CC terms that met our enrichment criterion of pFDR≤0.1 can be found in Dataset S3.
(PDF)

Figure S2 **Paired differences between the standard deviation of genes in children vs. adults.** The boxplot is made from for all 1020 probes with highest variance across all samples in the postmortem dataset. Positive values indicate larger standard deviation in the younger group (children <15 years), whereas negative values indicate larger standard deviation in the older group (adults ≥15 years). The median (heavy black line) represents the point at which 50% of the data are greater than (above the line) or less than (below the line) this value. The upper quartile (open box above the median) represents the 25% of the data greater than the median. The lower quartile (open box below the median) represents the 25% of the data less than the median. Note that 78% of the probes have greater standard deviation in the younger group. The maximum (above the upper quartile) and minimum (below the lower quartile) values excluding outliers are also shown. Outliers are drawn as open circles.
(PDF)

Table S1 **Sample information for all individuals.**
(XLSX)

Dataset S1 **Probes with greater variance in expression in children than in adults.**
(XLS)

Dataset S2 **GO and Kegg Pathway analyses for probes with greater variance in children than in adults using as reference all genes called present on the array.**
(XLS)

Dataset S3 **GO and Kegg Pathway analyses for probes with greater variance in children than in adults using as reference the top 5% of genes with highest variance.**
(XLS)

Dataset S4 **Genes found to be significant in variance analyses (great variance in children than adults) in both the Harris et al. 2009 (GEO accession: GSE13564) and current datasets.**
(CSV)

Dataset S5 **GO analysis for probes with greater variance in children than adults.** (Harris et al., 2009 Dataset; GEO accession: GSE13564).
(XLS)

Dataset S6 **Intersection of 302 genes with greater variance in children with Mammalian Genome Informatics dataset of Mammalian Phenotype ID: MP:0003631 and MP:0005386).**
(XLS)
