# Supplementary figures and images for: Dynamic Gene Expression in the Human Cerebral Cortex Distinguishes Children from Adults
Source: PLoS One. 2012 May 30;7(5):e37714. doi: 10.1371/journal.pone.0037714 (PMC3364291; doi:10.1371/journal.pone.0037714)

## Overrepresented GO Molecular Functions

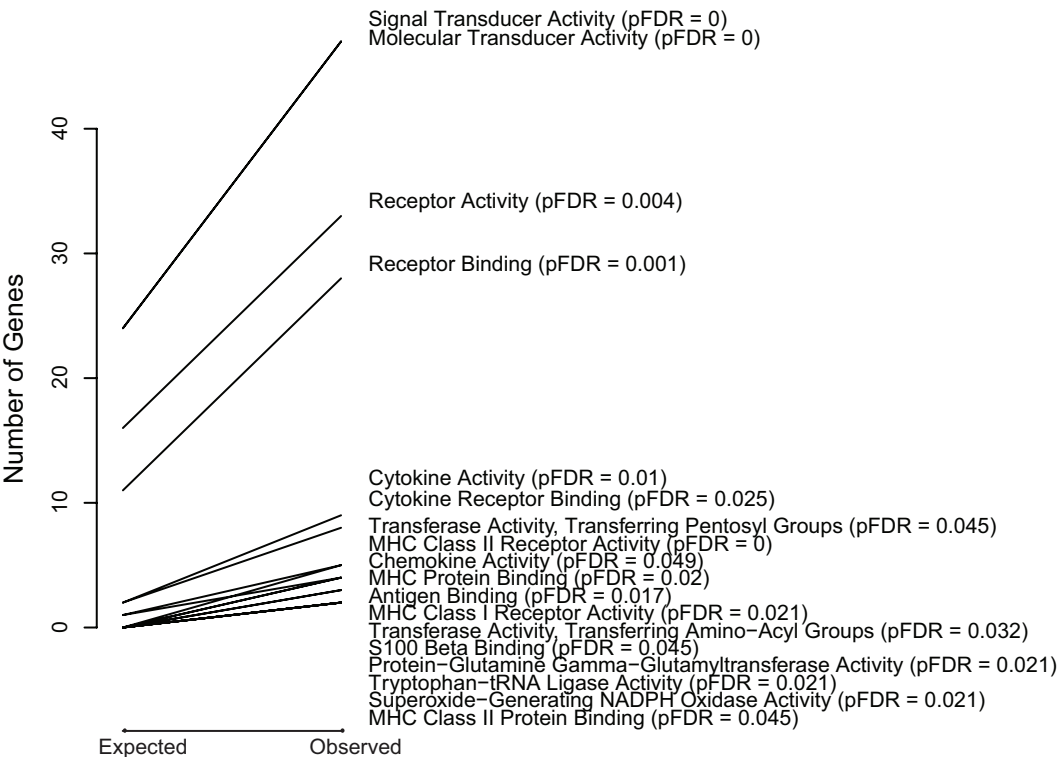

## Overrepresented GO Cellular Components

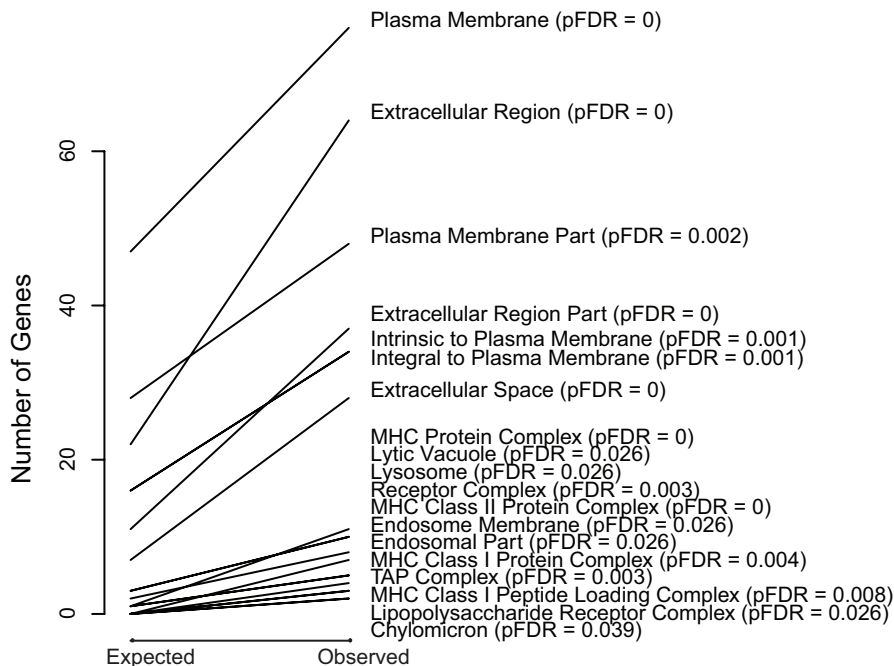

Supplement: Figure S1 — Gene Ontology analyses. GO term (Molecular Function and Cellular Component) analyses for probes with greater variance in childhood than in adulthood using as reference all genes called present on the array. The expected number of genes is the number of genes predicted for this term by random chance. The observed number of genes is the number of genes actually present in our dataset for this term. For example, in this context we would expect by random chance to see >1 gene annotated to the GO_MP term ‘MHC class II receptor activity’ (GO:0032395). Instead, we observed 5 genes annotated to this term (pFDR = 0). The steepness of the slope of each line reflects statistical significance with steeper lines having smaller pFDR values. Those categories with the greatest slope (pFDR = ≤0.05) are labeled in this figure. All 26 GO_MP terms and 30 CO_CC terms that met our enrichment criterion of pFDR≤0.1 can be found in Dataset S3. (PDF) [file pone.0037714.s001.pdf]

Paired SD Younger – SD Older

-0.5 0.0 0.5 1.0 1.5 2.0

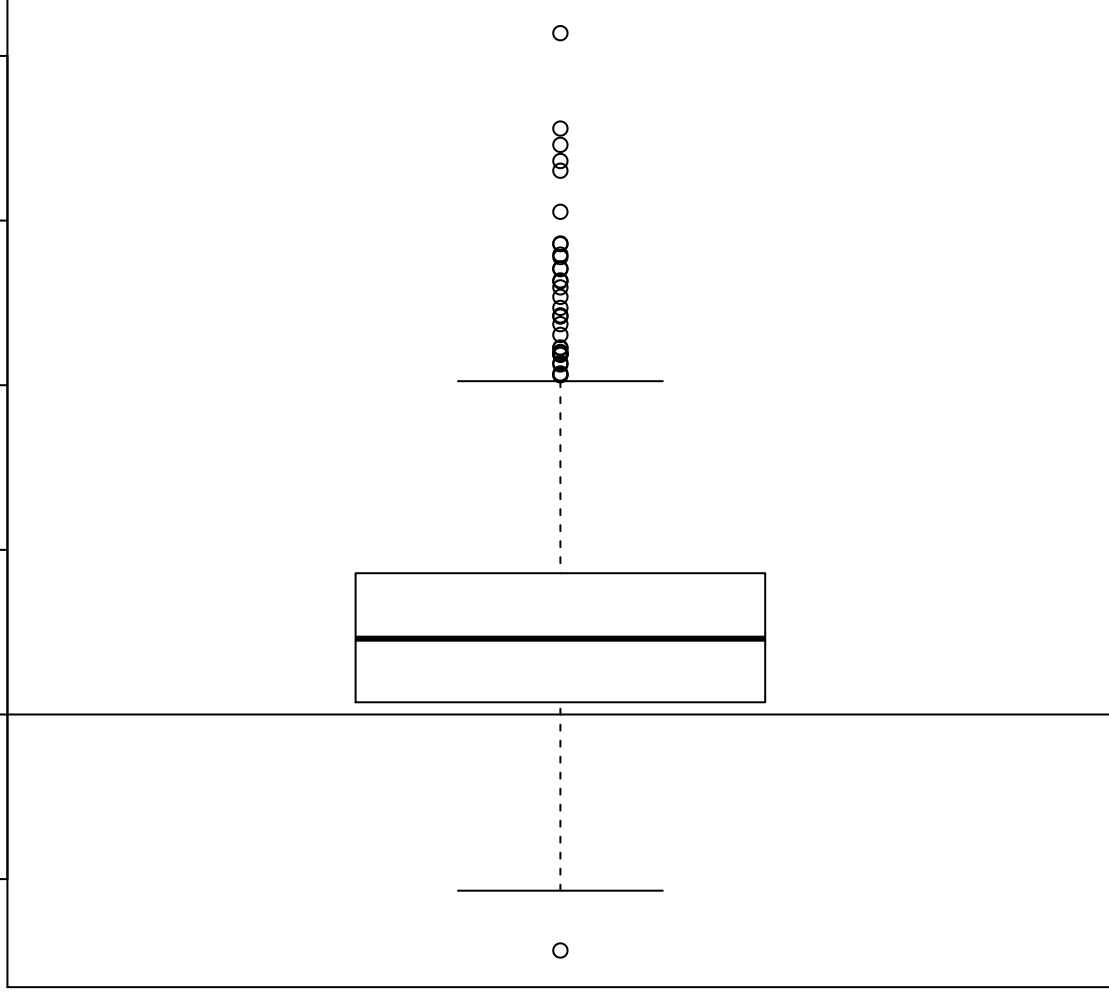

Supplement: Figure S2 — Paired differences between the standard deviation of genes in children vs. adults. The boxplot is made from for all 1020 probes with highest variance across all samples in the postmortem dataset. Positive values indicate larger standard deviation in the younger group (children <15 years), whereas negative values indicate larger standard deviation in the older group (adults ≥15 years). The median (heavy black line) represents the point at which 50% of the data are greater than (above the line) or less than (below the line) this value. The upper quartile (open box above the median) represents the 25% of the data greater than the median. The lower quartile (open box below the median) represents the 25% of the data less than the median. Note that 78% of the probes have greater standard deviation in the younger group. The maximum (above the upper quartile) and minimum (below the lower quartile) values excluding outliers are also shown. Outliers are drawn as open circles. (PDF) [file pone.0037714.s002.pdf]
